# Our genes, our selves: hereditary breast cancer and biological citizenship in Norway

**DOI:** 10.1007/s11019-017-9803-0

**Published:** 2017-09-22

**Authors:** Pål Møller, Eivind Hovig

**Affiliations:** 1 Department of Tumor Biology, Institute for Cancer Research, Oslo University Hospital, Oslo, Norway; 2 Department of Human Medicine, Universität Witten/Herdecke, Witten, Germany; 3 Research Group Inherited Cancer, Department of Medical Genetics, Oslo University Hospital, Oslo, Norway; 4 Primary investigator to the Prospective Lynch Syndrome Database, http://www.PLSD.eu/; 5 Member of the board, European Hereditary Tumour Group, https://ehtg.org/; 6 Department of Informatics, University of Oslo, Oslo, Norway; 7 Institute of Cancer Genetics and Informatics, Oslo University Hospital, Oslo, Norway

**Keywords:** Breast cancer, *BRCA1*, *BRCA2*, Inherited, Biological citizenship, Identity politics

## Abstract

The concept ‘hereditary breast cancer’ is commonly used to delineate a group of people genetically at risk for breast cancer—all of whom also having risk for other cancers. People carrying pathogenic variants of the *BRCA1* and *BRCA2* genes are often referred to as those having predisposition for ‘hereditary breast cancer’. The two genes, however, are when altered, associated with different risks for and dying from breast cancer. The main risk for dying for carriers of both genes is from ovarian cancer. These biological facts are of philosophical interest, because they are the facts underlying the public debate on *BRCA1/2* genetic testing as a model for the discussion of how to implement genetic knowledge and technologies in personalized medicine. A contribution to this public debate describing inherited breast cancer as ‘biological citizenship’ recently printed in *Med Health Care and Philos* illustrated how fragmented and detached from the biological and socio-political facts this debate sometimes is. We here briefly summarize some of the biological facts and how they are implemented in today’s healthcare based on agreed philosophical, ethical and moral principles. The suggestion of a ‘biological citizenship’ defined by hereditary breast cancer is incorrect and ill-advised. ‘Identity politics’ focusing hereditary breast cancer patients as a group based on a bundle of ill-defined negative arguments is well known, but is supported neither by scientific nor philosophical arguments. To those born with the genetic variants described, the philosophical rule of not doing harm is violated by unbalanced negative arguments.

In their report in *Med Health Care and Philos*, Solbrække et al. (2017) describe a reality which is interestingly different from the scientific and social reality known to us in Norway, and—in our opinion—cite a number of Norwegian papers referred in a way which may be misunderstood. The declared starting point and nomenclature are both without references and definitions. The introduction, methods and results seem to meet the notion that ‘you see only what you look for; you recognize only what you know’ (Sosman, http://emedsa.org.au/Students/Maxims.htm). Their reality is so different from ours that it is tempting to cite the current sociopolitical notion on ‘alternative facts’ (https://en.wikipedia.org/wiki/Alternative_facts). The paper refers to a conflict between the two major hospitals in Norway, but without describing what it is about. Moreover, it seems to consider Norway to be an isolated cultural system that is not integrated in Europe, and from a few selected patients’ statements in USA and Norway as Norway being detached from the cultural divergence between USA and EU when it comes to perception of freedom and autonomy.

On the contrary, Norway is integrated into the socio-cultural debate on how to use technology and resources available to those in need for health care in EU. In short, this is about how to disperse the available good to as many as possible within the resources available, and with the understanding that if someone exercises his/her ‘rights’ too much, it will deprive someone else from their needs. USA has the definition of freedom that anyone may do as they want to within their personal means, and where the individual has no or less responsibilities for their fellow citizens. For deeper clarification, see Public health genomics (http://www.phgen.eu/typo3/index.php), for relevance to current political discussion, see ‘we are not Denmark’ as stated in the recent USA presidential election campaign (https://www.commondreams.org/views/2016/02/26/we-are-not-denmark-hillary-clinton-and-liberal-american-exceptionalism). Both the EU and USA way of thinking claims to be right according to the Declaration of Human Rights, as specified in the Oviedo amendment with respect to genetics (Oviedo convention, http://www.coe.int/en/web/conventions/full-list/-/conventions/treaty/164). They both base their positions on the right of an individual to know whether he/she wants to know as to take part in decisions with impact on their own future. In the world-wide discussion, particularly on BRCA testing, the user organization Global Alliance for Genomic Health (http://genomicsandhealth.org/) agrees with those of us interpreting this as a right to be genetically tested for actionable disease predispositions, if one wants to. The two different ways of thinking, however, lead to two profoundly different patient attitudes: In the USA, it is accepted to exercise your autonomy within your means (‘my medical choice’ for a rich woman). In the EU, it is about what one person could rightfully and morally claim of the common resources to meet his or her personal needs. When Solbrække et al. cite two patients—one from USA and one from Norway—they seemingly do not understand that the two have these different positions, and that they both had been integrated into their social settings and rehearsed by their environments to perform accordingly. In addition, the Norwegian patient’s statement was filtered through a journalist obliged to relate to these norms. In the Declaration of Human Rights, autonomy and the ‘right to know’ are core philosophical arguments and values, but they are completely overlooked by Solbrække et al.

Solbække et al.’s paper claims there is a medical condition defined as ‘hereditary breast cancer’—which is a fiction: There is no such inherited disorder identified. All forms of hereditary breast cancers are part of inherited multiorgan cancer syndromes. They overlook the clinically recognized inherited Li-Fraumeni syndrome, in which breast cancer is a lethal manifestation, and they overlook Cowden syndrome, where breast cancer is cured. They focus *BRCA1/2* associated breast cancer, and make the mistake of considering the two very different forms of breast cancer associated with *BRCA1* and *BRCA2*, respectively, to be biologically similar disorders. They are not. They are different in every biological way of describing the tumors. The basic recommendation of all carriers of *BRCA1* */* *2* pathogenic variants in Norway is follow-up aiming at early diagnosis and cure, and this is provided to all carriers identified. Solbrække et al. fail to mention that *BRCA2*-associated breast cancer is usually cured if diagnosed by today’s methods for early diagnosis (Evans et al. 2016), while about two-thirds of *BRCA1*-associated breast cancer cases may be cured this way (Tharmaratnam et al. 2014). Prophylactic mastectomy is in Norway recommended for neither, but it is an option that the patient may choose after having been fully informed and consented in writing, so as to avoid the ‘journey of being diagnosed with and treated for cancer’ and the risk of dying, despite that follow-up aiming at early diagnosis and cure has been undertaken.

When detected outside of the dedicated follow-up programs, the prognosis is bad for breast cancer in both *BRCA1* and *BRCA2* carriers. When we had to decide on the above, the Norwegian social debate was different from that in the other EU countries and in the USA: Geneticists in Norway advocated all *BRCA1/2* carriers to be included in follow-up to learn whether or not prophylactic mastectomy was needed to avoid dying from breast cancer. In consequence, we aggregated information to produce the knowledge mentioned above. The basic question is whether or not the patients should be informed and have the choice. The alternative is that the patients should not be informed and/or not have the choice. Which is not discussed by Solbrække et al. Are all the arguments presented by Solbrække et al. to be interpreted as they are against informing the patients? Or against providing them the choices mentioned? What do they mean with ‘the sheer absurdity of being invited to consider choices relating to aesthetics’—are they against the patients to be informed for them to take part in decisions on treatment modalities when diagnosed with cancer? Do they deny body self-image perception to be an argument patients may use? Don’t they know that many patients are relating to such arguments when selecting treatment modalities, and do they not understand that the patients they cite in their paper expressed positions on this? Are Solbrække et al. in conflict with the general philosophic platform for modern health care? Do they advocate a paternalistic system where the doctor decides, without telling the patient why? If so, they have a lot of philosophical explaining to do.

What Solbrække et al. do discuss, however, is the inherited breast-ovarian cancer syndrome (initially described by Paul Broca in 1866), in which most die from ovarian cancer. The *BRCA1/2* syndrome is not ‘inherited breast cancer’—it is two different multi-organ cancer syndromes where most deaths are caused by ovarian cancer. Despite all attempts on early diagnosis to improve cure, the majority of those who contract ovarian cancer will die from the disease. The international (Finch et al. 2014) recommendation for BRCA1/2 carriers is to remove the ovaries when childbearing is completed, which is what the Norwegian guidelines echo. Solbrække et al.’s focus on the breast and on mastectomy, which until recently few Norwegian patients selected, is unsubstantiated and in conflict with the facts of what the international and national guidelines say. From a biological perspective, the ovaries are the key organs determining female body functions. Solbrække et al.’s focus on the breasts is in keeping with an emotional, feministic, sexual debate, and they offer absolutely no explanation why they are so interested in the female breasts, and not the greater risk of dying from ovarian cancer.

Everything discussed above is based on genetic testing. The key question is who has access to genetic testing? But Solbrække et al. are not interested in this key question. They should have been, because that has been the basic difference between Norway and the rest of the world, and this difference is underlying the stories told by all their patients. From the start, everything was about family history—the volume of patients and precision in the arguments has increased, but there are in principle no new arguments to the discussion after the *BRCA* genes were identified (Moller et al. 1999). At that time, genetic testing became possible, but not available. Later, genetic testing became available for a few: in USA to those who could pay, in EU it became a social discussion who should get priority. Subsequently, it became available, but at a high cost, and from now onwards, the cost is no problem—it’s in principle available to all. But not in Norway, who has a special law on biotechnology making it illegal to provide predictive genetic testing to anyone not approved by the Government, abiding by some old and outdated risk calculations (https://lovdata.no/dokument/NL/lov/2003-12-05-100). This is a philosophical problem Solbrække et al. fail to mention, and it is the basis for all the Norwegian patients’ statements in their paper. Close to no other countries have adopted these Norwegian arguments, and many of our fellow geneticists abroad consider them violations of human dignity. Solbræbrække et al.’s interest is a few selected patients’ feelings outside of any of the contextual settings mentioned above. Having personally discussed these matters with some thousand patients, we can confirm that the selection of arguments brought forward by Solbrække et al. are disease-creating, by telling the patients how dreadfully difficult their lives are, and overlooking all the rest. The patients feel harmed by their position. Solbrække et al. are violating the philosophical rule of not doing harm, and they are not discussing the philosophical principle that doing nothing when you can do good is a deliberate action making you responsible for the consequences. And, as a gynecologist once said in a discussion on these matters: ‘life quality is difficult to measure when the patient is dead’.

What is philosophically new, but not discussed by Solbrække et al., is that their Norwegian references clearly state that family history as a selection criterion for whom to be *BRCA* tested is outdated. Most women with breast cancer in their families do not have a pathogenic *BRCA* variant, and most women with a pathogenic *BRCA* genetic variant do not have aggregation of breast cancer in their close relatives. The special philosophical issue on genetic testing has been the involvement of the family members disclosing sensitive medical information within the families, which is a violation of the privacy within the families, as a prerequisite to get health care. This is no longer needed, and there is no longer any argument to have any special rules for clinical genetics, as compared to other health care activities. Genetic testing can be and should be as private as any other health care. Genetic testing should be available for all who want it: this is the burning ethical, philosophical, moral, political and professional debate—but Solbrække et al. seem not to be aware of that. Fear of breast cancer because of family history is not a novelty, and the effect of *BRCA* testing is relieving the fear because most will not have a pathogenic *BRCA* variant. For those who do have a pathogenic variant, most professionals in life quality possibly agree with the notion that ‘it is better to know than to fear’.

There is nothing fundamentally new in genetic testing. Solbrække et al. may read the introduction in the Bible on the consequences of eating the fruit of knowledge: it includes no special mention on *BRCA* testing. We are blood-typing patients to avoid transfusion problems, Rh-testing pregnant women to avoid disease in the kids to come, HIV-testing to prevent AIDS, determining cholesterol blood levels to prevent cardiac disease, screening all adult women with mammography, vaccinating against infections, advocating malaria prevention in certain areas, advocating exercise and food advices to avoid obesity, discussing PSA testing elderly men, etc. You may visit our website (Lynch syndrome cancer risk, http://www.lscarisk.org/) to learn the risk for inherited Lynch syndrome, including intestinal, endometrial, ovarian and urological cancer, and the paper referenced by them, describing how we now cure most of the patients. The only interesting problem we find in Solbrække et al.’s paper is their interest in the female breasts, and which is an interest they have not explained. Further, the statement of Solbrække et al. of “Scientific discoveries of human DNA have led to the rapid commercialization of gene technology” seems in a Norwegian setting at best strange in the light of commercial DNA testing in Norway being legally prohibited.

Solbrække et al.’s paper fail to discuss the philosophical questions on how to define risk groups for health care, and how to prevent disease in public health strategies. Their delineation of ‘inherited breast cancer’ is genetically erroneous. They have misunderstood the concept of biological citizenship. What they discuss are opportunistic highly selected arguments for a group constructed on sociopolitical, sexual, and emotional reasons—a discussion commonly referred to as identity politics (Identity politics, https://en.wikipedia.org/wiki/Identity_politics). To us, their paper is not scientifically valid.
